# Trans-Umbilical Lymphadenectomy Using an Articulating Bipolar Vessel-Sealing Device (TULAB) during Robotic Surgery for Gastric Cancer: Enhancing the Surgeon’s Eye for Reduced-Port Robotic Gastrectomy

**DOI:** 10.3390/cancers15225371

**Published:** 2023-11-11

**Authors:** Raeyoon Jeong, Min-Se Kim, Chang-Min Lee, In-Young Lee, Sungsoo Park, Seong-Heum Park

**Affiliations:** 1 Department of Surgery, Korea University College of Medicine, Seoul 02841, Republic of Koreabeareatbear@nate.com (M.-S.K.); irene.inyoung.lee@gmail.com (I.-Y.L.); kugspss@korea.ac.kr (S.P.); pshchw@korea.ac.kr (S.-H.P.); 2 Department of Surgery, Korea University Medical Center Ansan Hospital, Ansan 15355, Republic of Korea; 3 Department of Surgery, Korea University Medical Center Anam Hospital, Seoul 02841, Republic of Korea

**Keywords:** gastric cancer, reduced-port robotic distal gastrectomy (RRDG), Vessel Sealer Extend^®^ (VSE), trans-umbilical lymphadenectomy using an articulating BVSD (TULAB)

## Abstract

**Simple Summary:**

To facilitate performing a lymphadenectomy during a reduced-port robotic distal gastrectomy for gastric cancer, we developed Vessel Sealer Extend^®^ (Intuitive Surgical, Sunnyvale, CA, USA), a bipolar vessel-sealing device with an articulating jaw. Using the Vessel Sealer Extend^®^ (Intuitive Surgical), we performed trans-umbilical lymphadenectomy using an articulating bipolar vessel-sealing device and found that reduced-port robotic distal gastrectomy with trans-umbilical lymphadenectomy using an articulating bipolar vessel-sealing device had similar outcomes to conventional laparoscopic distal gastrectomy in terms of the incidence of postoperative morbidity and the number of harvested lymph nodes. In addition, because reduced-port robotic distal gastrectomy is associated with fewer incisions, intra-abdominal adhesions can be minimized.

**Abstract:**

Background: Docking the scope and instruments through a multi-channel trocar has enabled reduced-port robotic distal gastrectomy (RRDG) for gastric cancer. To facilitate lymphadenectomy over the anatomical hindrances during RRDG, we recently introduced the Vessel Sealer Extend^®^ (VSE) (Intuitive Surgical, Sunnyvale, CA, USA), a bipolar vessel-sealing device (BVSD) with an articulating jaw. Methods: From May 2020 to August 2023, we performed RRDG to treat T1 gastric cancer. One endoscope arm and three instrument arms of the da Vinci^®^ Xi Surgical System (Intuitive Surgical) were used. During the lymphadenectomy, the endoscope and VSE (Intuitive Surgical) were docked through a multi-channel trocar established on a trans-umbilical incision. Two Cardiere forceps were docked through cannulas established on each flank. A trans-umbilical lymphadenectomy using an articulating BVSD (TULAB) was then performed. Results: A total of 42 patients underwent planned RRDG with the TULAB technique. The number of retrieved lymph nodes did not differ between the patients who underwent RRDG and those who underwent conventional laparoscopic distal gastrectomies (CLDG) (*p* = 0.362). There was no statistically significant difference in postoperative complications between the RRDG and CLDG group (*p* = 0.189). The mean time to first semi-fluid diet was shorter in the patients who underwent RRDG than CLDG (*p* = 0.030), and the incidence of postoperative ileus was lower in the RRDG group than the CLDG group (0% and 9.9%, respectively, *p* = 0.034). Conclusions: Despite use of fewer ports, RRDG with TULAB had similar outcomes to CLDG in terms of the incidence of postoperative morbidity and the number of harvested lymph nodes. Furthermore, by reducing the number of incisions, the incidence of the intra-abdominal adhesions can potentially be lowered when RRDG is used.

## 1. Introduction

Although robotic gastrectomy is known for its operation times and higher costs than other surgical procedures for gastric cancer, the articulating devices and tremor-filtering function provided by the robotic surgical system have several advantages in gastric cancer surgery [1,2,3]. In particular, robotic instruments have been noticed by surgeons who pursue reduced-port gastrectomy for gastric cancer because the articulation of the devices can help to avoid collisions between the instruments during reduced-port surgery. 

Several studies have shown the effects of articulation in reduced-port robotic distal gastrectomy (RRDG) for gastric cancer [4,5,6,7,8,9,10,11]. Although there are differences in the strategies used to achieve the surgical goal through the reduced ports, all these reports have suggested the advantages associated with “traction” in using the articulating instruments provided by the robot surgical system, which include (1) traction of the tissues behind the vessels or organs (e.g., supra-pancreatic lymphadenectomy), (2) traction of the tissues with less frequent collision (e.g., supra-duodenal trimming), and (3) traction of tissues hidden in a ‘pit’-like space (e.g., lymph node no. 11p or 12a). 

However, these features have been emphasized in conventional robotic surgery as well as RRDG. Therefore, to improve the feasibility of RRDG procedures, other aspects that can be enhanced by the articulating function need to be explored. An articulating vessel-sealing device has not previously been used for lymphadenectomy, despite its clear advantages of articulating function. 

Of the articulating energy devices intrinsic to robotic surgical systems, we focused on the Vessel Sealer Extend^®^ (VSE) (Intuitive Surgical) for lymphadenectomy during RRDG, as its articulating tip has been shown to be effective in narrow spaces such as the surgical field of an endoscopic thyroidectomy [12]. This could help to overcome collisions within the multi-channel trocar during RRDG. However, VSE has not replaced the ultrasonic energy device in previous RRDG procedures (Table 1). The ultrasonic energy device does not contribute to reducing the number of ports in developing RRDG procedures; it needs to be inserted through a separate cannula due to its stiff shaft and water-splashing phenomenon [12,13]. Nevertheless, the ultrasonic energy device has been steadily adopted in RRDG because its cavitation effect significantly facilitates lymphadenectomy by providing insight into the surgical plane. This is a primary reason why the surgeons who are developing RRDG hesitate to depend on the unfamiliar mechanism of VSE during a lymphadenectomy, since VSE does not provide the cavitation effect. 

We designed this study to validate the safety of RRDG using VSE. We provide the technical details of RRDG using VSE and compare clinical outcomes between patients who underwent RRDG using VSE and a conventional laparoscopic distal gastrectomy (CLDG) for gastric cancer.

## 2. Methods

### 2.1. Patients

This study was a retrospective cohort study performed in a single institution. We reviewed the electronic medical charts of consecutive patients clinically diagnosed with gastric adenocarcinoma who underwent robotic distal gastrectomy at the Korea University Medical Center Ansan Hospital between May 2020 and August 2023. These patients provided written informed consent for participation in all the procedures associated with RRDG using VSE. Approval to perform research on human subjects in this study was provided by the Institutional Review Board of Korea University Medical Center Ansan Hospital (registration number: 2023AS0184).

### 2.2. Eligibility Criteria

For study enrollment, the patients were required to meet the following criteria:

(i) Histologically proven gastric adenocarcinoma
(ii) Age 20–80 years
(iii) Clinical stage I gastric cancer based on the 8th edition of the American Joint Committee on Cancer staging system [14] (clinical stage was determined based on the findings of gastrofiberscopy and abdominal computed tomography)
(iv) Appropriate candidate for R0 surgery using a distal gastrectomy with D1+ or D2 lymphadenectomy
(v) American Society of Anesthesiology (ASA) score of I, II, or III
(vi) Eastern Cooperative Oncology Group performance status of 0 or 1
(vii) Scheduled for robotic surgery

### 2.3. Surgical Procedures

All surgical procedures were performed by one surgeon (C.M.L.), who had performed approximately 150 reduced-port laparoscopic gastrectomies for gastric cancer, including approximately 60 single-port laparoscopic gastrectomies and 30 robotic gastrectomies, before he began RRDG using VSE. The da Vinci^®^ Xi Surgical System (Intuitive Surgical) was used for lymphadenectomy in all the patients.

#### 2.3.1. Preparation for the Console Period (Docking of the Robotic Surgical System)

**Figure 1:**
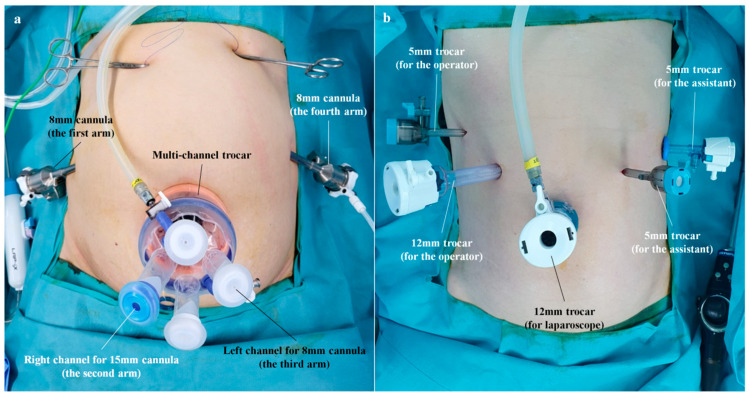
Port locations on the abdomen. (**a**) Location of the ports in a reduced-port robotic distal gastrectomy (the robot arm docked to each port is indicated in parentheses). (**b**) Location of the ports in a conventional laparoscopic distal gastrectomy.

In the operating room, the patient was placed on the table with both legs abducted under general anesthesia. The operating table was adjusted to create a reverse Trendelenburg position. After a 30 mm transumbilical incision was made on the patient’s abdomen, a commercial multi-channel trocar, Gloveport^®^ (Nelis, Bucheon, Republic of Korea) was inserted through the transumbilical incision using the Hasson technique [15]. After a pneumoperitoneum was created using carbon dioxide at a pressure of 15 mmHg, a binocular lens endoscope with a 30° downward view was inserted through the ‘left’ channel of the Gloveport^®^ (Figure 1a). Under the guidance of the endoscope, one 8 mm straight cannula was placed along the right flank, and another 8 mm straight cannula was inserted along the left flank. Then, one 15 mm straight cannula (Intuitive Surgical) was inserted into the ‘right’ channel of the Gloveport^®^ (Figure 1a). Following placement of the cannulas, the robotic cart was positioned beside the patient. Then, docking was performed in the following order: (1) the third arm was docked to the cannula inserted in the ‘left’ channel of the Gloveport^®^, (2) the first arm was docked to the cannula of the right flank, (3) the fourth arm was docked to the cannula of the left flank, and (4) the second arm was docked to the cannula inserted in the ‘right’ channel of the Gloveport^®^. Under the guidance of the endoscope inserted via the ‘left’ channel of the Gloveport^®^, Cadiere forceps (Intuitive Surgical) were inserted through the cannula of the left flank. VSE (Intuitive Surgical) was then introduced through the cannula inserted into the ‘right’ channel of the Gloveport^®^. Finally, another pair of Cadiere forceps was inserted through the cannula of the right flank. Cadiere forceps were substituted with Maryland bipolar forceps (Intuitive Surgical) to achieve sharp dissection. The docking status of each arm is shown in Figure 1a.

#### 2.3.2. Lymphadenectomy in Lymph Nodes 4sb, 5, and 6

**Figure 2:**
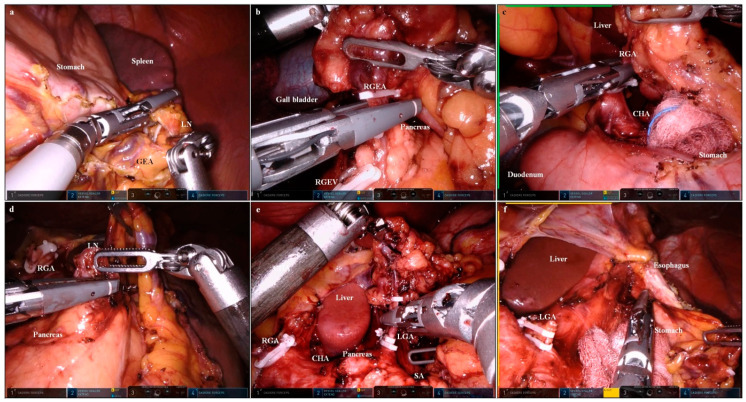
Images of a lymphadenectomy using the Vessel Sealer Extend^®^ (Intuitive Surgical, Sunnyvale, CA, USA). (**a**) Lymphadenectomy in lymph node 4sb (GEA, gastro-epiploic arcade; LN, lymph node); the greater curvature side of the gastric fundus was cleared using VSE (second arm), with the stomach pulled using Cadiere forceps (first arm). (**b**) Lymphadenectomy in lymph node 6 (RGEA, right gastro–epiploic artery; RGEV, right gastro-epiploic vein); the right gastro–epiploic artery was divided using VSE (second arm), with the duodenum pulled cranially using Cardiere forceps (first arm). (**c**) Lymphadenectomy in lymph node 5 (CHA, common hepatic artery; RGA, right gastric artery); the right gastric artery was divided using VSE (second arm), with the duodenum pulled caudally using Cardiere forceps (first arm). (**d**) Lymphadenectomy in lymph node 8 (LN, lymph node; RGA, right gastric artery); the supra-pancreatic lymph node was dissected using VSE (second arm), with the left gastric pedicle pulled cranially using Cardiere forceps (first arm). (**e**) Lymphadenectomy in lymph node 7 (CHA, common hepatic artery; LGA, left gastric artery; RGA, right gastric artery; SA, splenic artery); the left gastric artery was divided using VSE (second arm), with the left gastric pedicle pulled cranially using Cardiere forceps (first arm). (**f**) Lymphadenectomy in lymph node 1 (LGA, left gastric artery); the lesser curvature side of the esophago–gastric junction was cleared using VSE (second arm), with the left lateral section of the liver pushed cranially using Cadiere forceps (first arm).

The falciform ligament and left lobe of the liver were raised in the cephalad direction using combined suture retraction [16]. A D1+ or D2 lymphadenectomy for a curative distal gastrectomy was then performed based on the Japanese Gastric Cancer Treatment Guidelines 2014 (ver. 4) [17]. When the named vessels were ligated, the VSE inserted in the ‘right’ channel of the Gloveport^®^ (docked on the second arm) was replaced with a Hem-o-lok applier (Intuitive Surgical). To keep the surgical field clean, sterile gauze was inserted and removed through the empty channels (not docked to the robot arm) of the Gloveport^®^. Figure 2a–c are images of a lymphadenectomy in lymph nodes 4sb, 6, and 5, respectively. After ligation and division of the right gastric artery, the VSE was released from the second arm. 

#### 2.3.3. Division of the Duodenum

**Figure 3:**
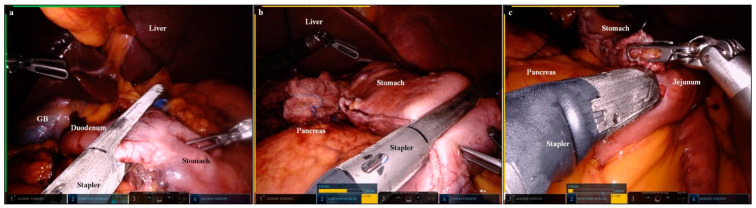
Images of robot-controlled surgical stapling. (**a**) The duodenum was divided using the robot-controlled surgical stapler (second arm) (GB, gall bladder). (**b**) The stomach was divided using the robot-controlled surgical stapler (second arm). (**c**) A gastro–jejunal anastomosis was performed using the robot-controlled surgical stapler (second arm).

A robotic linear stapler (Intuitive Surgical) was inserted into the 15-mm cannula (‘left’ channel of the Gloveport^®^) docked to the second arm. Then, the duodenum was divided using the robotic-controlled surgical stapler (Figure 3a). Following division of the duodenum, the robotic-controlled surgical stapler was released from the second arm.

#### 2.3.4. Lymphadenectomy in Lymph Nodes 7, 8, 9, 11p, and 12a

The VSE was inserted into the ‘right’ channel of the Gloveport^®^ (docked to the second arm). A lymphadenectomy was performed in the sequence of lymph nodes 8, 9, 12a, 7, 11p (Figure 2d–f). 

#### 2.3.5. Lymphadenectomy in Lymph Node 1

With the left lateral section of the liver retracted using the Cadiere forceps of the first arm, a lymphadenectomy was performed in lymph node 1. After the lymphadenectomy was complete, we exchanged the VSE for a robotic-controlled surgical stapler in the second arm. Then, the stomach was divided using the robotic-controlled surgical stapler (Figure 3b).

#### 2.3.6. Gastrointestinal Reconstruction

An anti-peristaltic gastrojejunal anastomosis was performed using the robotic-controlled surgical stapler (Figure 3c). The common entry hole of the stapling was closed using a barbed suture material. Following reconstruction, two closed drains were introduced through the cannula insertion wounds on the right and left flanks.

### 2.4. Core Interventions of RRDG Using VSE

(i) Usage of only VSE (Intuitive Surgical) as an energy device for the lymphadenectomy
(ii) Trans-umbilical manipulation of the energy device for the lymphadenectomy
(iii) Trans-umbilical manipulation of the robot-controlled surgical stapler for resection and reconstruction

### 2.5. Data Collection

Demographic data, including age, sex, body mass index (BMI), and ASA score, were collected from all the enrolled patients. In addition, clinical outcomes, including operation time, conversion to open or laparoscopic surgery, postoperative hospital stay, time to the first semi-fluid diet, and postoperative complications, were investigated. Postoperative complications were categorized based on the Clavien–Dindo classification of surgical complications [18].

We also investigated pathologic results, including tumor depth and numbers of retrieved and metastatic lymph nodes. 

### 2.6. Comparison of Clinicopathologic Outcomes to Internal Controls

Patients who underwent CLDG using an articulating laparoscopic grasper (ArtiSential; LivsMed, Seongnam, Republic of Korea) and articulating laparoscopic dissector (ArtiSential; LivsMed) for gastric cancer between May 2020 and August 2023 were considered internal controls. The location of ports is shown in Figure 1b.

To validate the clinical effectiveness of our RRDG procedure, the clinicopathologic outcomes of patients who underwent RRDG were compared to those of internal controls; at the initiation of this study, we stated that the following variables should be equivalent between the patients who underwent RRDG and CLDG: (1) postoperative complication rate and (2) number of retrieved lymph nodes.

### 2.7. Statistics

The continuous variables are presented as means (± standard deviations). Statistical analyses were performed using the chi-square test for categorical variables and Student’s *t* tests for continuous variables. A *p*-value threshold of 0.05 was considered statistically significant. All the statistical analyses were performed using R software (R Foundation for Statistical Computing, Vienna, Austria; http://cran.r-project.org/, accessed on 1 September 2023).

## 3. Results

Between May 2020 and August 2023, a total of 42 patients underwent RRDG for gastric cancer (Table 2), in which lymph node dissection (LND) was performed using VSE.

### 3.1. Patient Demographics

The patient demographics are shown in Table 3. The mean age of the enrolled patients was 55.3 ± 10.3 years, and the mean BMI was 24.6 ± 3.1 kg/m^2^. 

### 3.2. Clinicopathologic Outcomes

A D1+ or D2 LND was performed using VSE in every patient. No patient underwent conversion to open surgery or laparoscopic surgery. 

The mean operation time was 275.2 ± 34.0 min, the mean hospital stay was 13.0 ± 4.1 days, and the mean time to the first semi-fluid diet was 4.0 ± 0.5 days (Table 4).

Nine patients (22.5%) experienced postoperative morbidities. Of these, two cases (4.8%) corresponded to Clavien–Dindo grade IIIa morbidities, namely fluid collection around the body of the pancreas. Both patients recovered without re-operation. None of the complications led to mortality (Table 4). 

According to the final pathologic reports, the mean number of retrieved lymph nodes was 42.6 ± 17.2. The mean number of retrieved lymph nodes did not differ between the 131 internal controls (patients who underwent CLDG) and the 42 patients who underwent RRDG (*p* = 0.362). The mean time to the first semi-fluid diet was significantly shorter in the RRDG group than the CLDG group (*p* = 0.030), while postoperative ileus was more frequent in the CLDG group than the RRDG group (9.9% vs. 0%, *p* = 0.034) (Table 4).

## 4. Discussion

In a previous study, we performed lymphadenectomy using two instrument arms to facilitate RRDG [7]. This procedure was designed using the concept of a reduced-port laparoscopic gastrectomy in which no assistant surgeon participates. The use of two instrument arms in lymphadenectomies can reduce the number of port incisions and help to avoid collision between robotic instruments.

However, performing an LND using two instrument arms carries some limitations; first, a robotic endoscope provides a narrower view than a laparoscope, and the operator may experience difficulty in the “unaided” procedure. Although the surgeon can choose the appropriate point for tissue traction during a reduced-port laparoscopic surgery in which there is bi-directional traction for the lymphadenectomy, it is difficult to achieve the organ positioning required to select the “key” traction point during reduced-port robotic surgery. This phenomenon became obvious when we attempted LND using two instrument arms in obese patients. In addition, several types of intra-abdominal self-traction instruments (i.e., free jaw clip or port-free endocavity retractor) are not compatible with robot surgical instruments. Thus, we could not realize the circumstances resembling those of conventional laparoscopic surgeries in which the assistant surgeon helps spread the targeted tissue during a lymphadenectomy.

For these reasons, we decided to use three instrument arms during the lymphadenectomy. To add an additional instrument arm without an additional incision, it was necessary to adopt another strategy to realize RRDG.

Therefore, we considered a bipolar vessel-sealing device (BVSD) as a solution for performing lymphadenectomies in RRDG, as recent robot systems come equipped with articulating BVSDs. In particular, VSE has sufficiently evolved to become more appropriate than the previous version of robotic BVSDs; its tip is thinner, and its joint occupies a more distal location. Furthermore, the activation mechanism of BVSDs is suited for performing lymphadenectomies in RRDG. As described in our previous report, BVSDs generate less fumes than ultrasonic energy devices, and it is feasible to insert the laparoscope and BVSD simultaneously into the rim of a multi-channel trocar. The lens of the laparoscope is not significantly affected by activation of the BVSD, even though the laparoscope and BVSD are in close proximity [13]. Inspired by our expertise using a BVSD for lymphadenectomies, we designed a new form of RRDG, in which the VSE and endoscope are inserted simultaneously into the cannulas that were established in a multi-channel trocar (port-in-port fashion). The operator’s line of sight should follow the point of lymphadenectomy unless the endoscope is affected by the VSE. Articulation of the VSE might contribute to fewer collisions than the straight shaft of an ultrasonic energy device. 

Simultaneous insertion of the endoscope and energy device into the rim of a multi-channel trocar is similar to the instrumental arrangement of a single-port laparoscopic gastrectomy (SPLG) performed using a multi-channel trocar. Because our center has accumulated expertise in SPLG [7,13,19,20,21], this situation was familiar and did not require a learning curve. Moreover, it is more feasible to perform trans-umbilical lymphadenectomy using an articulating BVSD (TULAB) than lymphadenectomy using a non-articulating BVSD during SPLG. 

At the beginning of the procedure, lymph node 4sb was the farthest field from the endoscope, and the stomach was retracted using Cardiere forceps. This instrument should be placed on the first arm docked in the right flank cannula, since the farthest instrument (from lymph node 4sb) can effectively retract the stomach with minimal disturbance. Here, we did not experience a collision in the TULAB procedure, as we achieved a near parallel arrangement of the endoscope and VSE. Furthermore, despite the close distance of the target to the instruments, collisions could be avoided by taking advantage of the articulating function of the VSE. In addition, as described above, the TULAB technique rarely results in visual disturbances, even when the VSE is close to the endoscope during LND. The VSE acts differently from the ultrasonic energy device by which the tissue fluids are spattered with the turbulence flow.

As a result, the TULAB procedure enabled us to realize RRDG via a single umbilical port and two flank cannulas (Figure 1a). In this study, RRDG showed similar short-term outcomes to those of CLDG in terms of most items other than the operation time (Table 4). Unfamiliarity with the TULAB technique might be one reason why the operation time of RRDG was longer than that of CLDG. Nevertheless, this new type of RRDG offers promising aspects.

**Figure 4:**
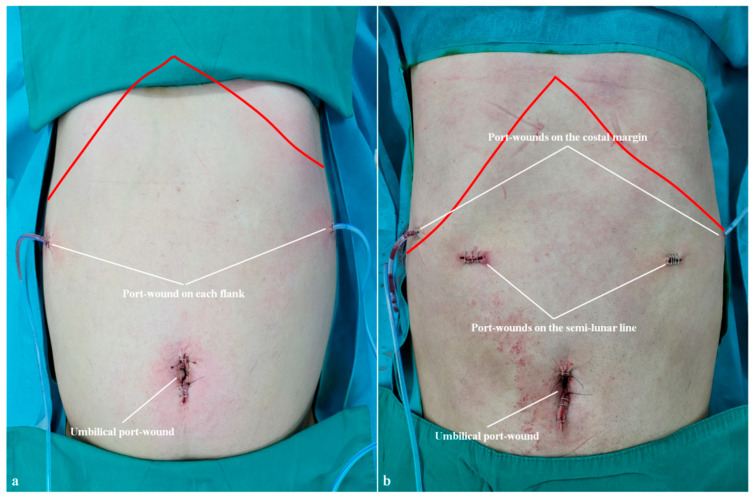
Port wounds on the abdomen. (**a**) Location of port wounds in a reduced-port robotic distal gastrectomy (red line indicates the costal margin). (**b**) Location of port wounds in a conventional laparoscopic distal gastrectomy (red line indicates the costal margin).

First, as shown in Table 4, the patients who underwent RRDG had a significantly shorter time to postoperative diet initiation than the CLDG patients. Inspired by this difference, we additionally compared the incidence of postoperative ileus between the two groups and found that RRDG was associated with a lower incidence of postoperative ileus than CLDG. This is likely due to differences in the location of port wounds between the two procedures (Figure 4). Although the location of the umbilical port wound is similar in the two procedures, CLDG requires a port wound in each semi-lunar line, whereas RRDG does not. Furthermore, the lateral port wound is closer to each flank in RRDG than in CLDG, while the lateral port wound is attached to each rib margin in CLDG but is separate from each rib margin in RRDG. Postoperative adhesions might be related to the presence of semi-lunar line port wounds and the location of the lateral port wounds; however, further study is needed to confirm this hypothesis.

**Figure 5:**
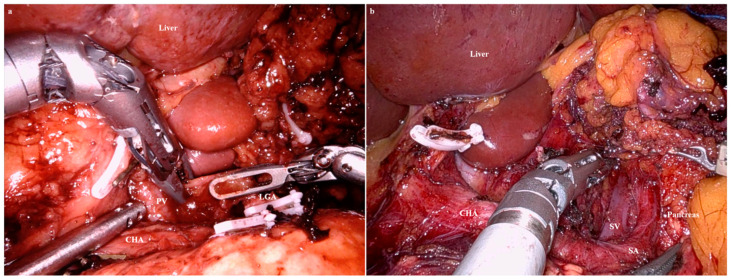
Exposure of the portal vein and the splenic vein during a lymphadenectomy. (**a**) The articulating bipolar vessel-sealing device (BVSD) was advantageous during the lymphadenectomy in lymph nodes 8 and 12a. (**b**) Due to the absence of a cavitation effect, it was difficult to perform a trans-umbilical lymphadenectomy using the articulating BVSD in lymph node 11p.

Another promising aspect of our new RRDG procedure is the potential to achieve a D2 lymphadenectomy with reduced port wounds. Although some surgeons have argued that the articulating point of the VSE is too proximal to provide a significant advantage over a straight device, use of the articulating BVSD appeared beneficial during LND for lymph nodes 8a and 12a. We performed effective LND using articulation of the VSE, and the portal vein was exposed in every case (Figure 5a). This was one of the important outcomes of our study, in which, to acquire the legitimacy of RRDG in patients with advanced gastric cancer (AGC), we investigated whether the splenic vein or portal vein could be exposed.

However, it was challenging to expose the splenic vein (Figure 5b), possibly due to the following limitations of the VSE.

The activating jaws of the VSE are thicker than those of ultrasonic energy devices or laparoscopic BVSDs, hindering delicate lymphadenectomy. In this study, we attempted to overcome this limitation of the VSE, but the thick jaws acted as a hindrance to LND in the slit-like space. Furthermore, in conditions where the thick jaws hid the surrounding structures, the articulating function could create an unexpected injury. In one case, during an LND in lymph node 11p, the splenic artery was injured by excessive angulation of the VSE. In addition, although the activation mechanism of the BVSD can help preserve a clean view during LND, absence of a “cavitation” effect might confuse novice surgeons during lymphadenectomy using the VSE. In particular, it was more difficult to expose the splenic vein than the portal vein, since the former runs under the meandering pathway of the splenic artery. In other words, without the cavitation effect, the depth of the splenic vein could not be determined. 

## 5. Conclusions

Despite a reduced number of ports, performing an RRDG with the TULAB procedure had similar short-term safety and oncologic outcomes to a CLDG. Moreover, the incidence of postoperative ileus was lower in patients who underwent an RRDG than those who underwent a CLDG, most likely due to the reduction in number of wounds in the former and fewer adhesions during the postoperative period. Although the operation time for the TULAB technique is longer than that for a conventional lymphadenectomy, the articulating function of the VSE is helpful in performing a D2 LND; using VSE, we were easily able to complete the LND despite the anatomical obstacles. However, VSE does not cause the cavitation effect, and it has a thicker jaw than ultrasonic energy devices, resulting in some difficulties with the LND in lymph node 11p. Regarding these issues, we expect that more advanced versions of robotic BVSDs will allow for successful D2 lymphadenectomies in patients with AGC. Robotic gears are continuously evolving; for instance, the VSE is an improvement over the initial version of the robotic BVSD. Additionally, if an articulating BVSD can be supported by a flexible endoscope, the range of a lymphadenectomy can be extended to secure oncologic safety in patients with AGC. 

## Figures and Tables

**Table 1:** Comparison of strategies to avoid inter-instrumental collisions in RPDG procedures.

	Number of Incisions	Location of Ports	Strategy to Avoid Collisions	Energy Device for LND	Limitations
Lee et al. (2017) [4]	3	1 SSP on umbilicus, 1 port on right flank, and 1 port (for the assistant) on left flank	Endoscope and two curved instruments were inserted via SSP	Ultrasonic energy device	No articulating function of energy device, difficulty in manipulating the two curved instruments
Seo et al. (2018) [5]	3	1 SSP on umbilicus, 1 port on right upper abdomen, and 1 port on left lower abdomen	Endoscope and one curved instrument were inserted via SSP	Ultrasonic energy device	No articulating function of energy device
Kim et al. (2020) [7]	3	1 GP on umbilicus and 2 ports on both flanks	Only the endoscope was inserted via GP	Ultrasonic energy device	No articulating function of energy device, no assistance
Choi et al. (2023) [11]	2	1 SSP on umbilicus, 1 port on right flank	Endoscope and two curved instruments were inserted via SSP	Ultrasonic energy device	No articulating function of energy device, difficulty in manipulating the two curved instruments
TULAB *	3	1 GP on umbilicus and 2 ports on both flanks	Endoscope and energy device were inserted via GP	Vessel Sealer Extend^®^ (Intuitive Surgical)	No cavitation effect of energy device

* Method used in the current study. RPDG, reduced-port laparoscopic distal gastrectomy; LND, lymph node dissection; SSP, Single-site^TM^ port (Intuitive Surgical, Sunnyvale, CA, USA); GP, Gloveport^®^ (Nelis, Bucheon, Republic of Korea); TULAB, trans-umbilical lymphadenectomy using an articulating bipolar vessel-sealing device.

**Table 2:** Demographic data of the patients who underwent a reduced-port robotic distal gastrectomy.

Number	Age	Sex	BMI	ASA Score	Specific Condition
1	55	Male	23.4	2	
2	59	Female	23.2	2	
3	60	Male	21.4	2	
4	64	Male	20.3	3	
5	60	Female	30.4	2	
6	62	Male	29.6	3	
7	60	Male	29.7	2	
8	44	Female	28.1	2	Previous abdominal surgery
9	43	Male	18.8	2	
10	52	Female	23.3	2	
11	73	Male	27.9	3	
12	58	Female	20.1	2	Previous abdominal surgery
13	55	Female	20.3	2	
14	56	Male	24.2	2	
15	39	Female	26.5	2	
16	28	Female	19.9	2	
17	58	Male	25.1	2	
18	45	Male	27.5	2	
19	44	Female	21.0	2	Previous abdominal surgery
20	54	Male	24.8	2	
21	79	Male	26.1	2	
22	60	Male	23.2	2	
23	56	Female	24.7	2	
24	52	Male	26.9	2	
25	56	Male	26.5	2	
26	60	Male	25.5	2	Preoperative ESD
27	74	Female	19.3	2	
28	71	Female	26.7	2	
29	61	Female	26.1	2	
30	46	Male	27.2	2	
31	67	Male	23.7	3	Liver cirrhosis
32	61	Female	28.5	3	
33	57	Female	22.4	2	
34	43	Male	23.4	2	
35	56	Male	23.3	2	
36	43	Female	20.5	2	Previous abdominal surgery
37	60	Male	26.4	2	
38	54	Male	24.0	2	
39	51	Male	25.1	2	
40	47	Female	29.0	2	
41	62	Male	23.6	2	
42	37	Female	25.5	2	

BMI, body mass index; ASA score, score graded by the American Society of Anesthesiologists physical status classification; ESD, endoscopic submucosal dissection.

**Table 3:** Comparison of baseline characteristics between the RRDG and CLDG groups.

	RRDG (*n* = 42)	CLDG (*n* = 131)	*p*
Age (years) at operation *	55.3 ± 10.3	63.6 ± 11.7	0.192
Sex			0.276
Male	24 (57.1%)	87 (66.4%)	
Female	18 (42.9%)	44 (33.6%)	
BMI *	24.6 ± 3.1	26.6 ± 8.9	0.069
Previous history of abdominal surgery	4 (9.5%)	6 (4.6%)	0.232
Previous history of ESD	1 (2.4%)	7 (5.3%)	0.426
ASA score			0.080
1	0 (0.0%)	1 (0.8%)	
2	36 (85.7%)	86 (65.6%)	
3	6 (14.3%)	38 (29.0%)	
4	0 (0.0%)	6 (4.6%)	

* Variables are expressed as mean ± standard error. RRDG, the patients who underwent a reduced-port robotic distal gastrectomy; CLDG, the patients who underwent a conventional laparoscopic distal gastrectomy; BMI, body mass index; ESD, endoscopic submucosal dissection; ASA, American Society of Anesthesiologists.

**Table 4:** Comparison of the clinicopathologic outcomes between RRDG and CLDG.

	RRDG (*n* = 42)	CLDG (*n* = 131)	*p*
Operation time * (min)	275.2 ± 34.0	236.5 ± 47.9	0.005
Hospital stay * (days)	13.0 ± 4.1	14.7 ± 8.5	0.083
Time to the first semi-fluid diet * (day)	4.0 ± 0.5	4.2 ± 1.4	0.03
Number of retrieved lymph nodes *	42.6 ± 17.2	50.9 ± 19.7	0.362
T staging (%)			
T1a	20 (47.6%)	70 (53.4%)	0.323
T1b	18 (42.8%)	51 (38.9%)	
T2	2 (4.8%)	9 (6.9%)	
T3	0 (0.0%)	0 (0.0%)	
T4	2 (4.8%)	1 (0.8%)	
Morbidity (%)	9 (22.0%)	15 (13.3%)	0.189
Morbidity, C-D grade > II (%)	2 (4.8%)	7 (5.3%)	0.883
Incidence of post-operative ileus (%)	0 (0%)	13 (9.9%)	0.034

* Variables are expressed as mean ± standard error. RRDG, the patients who underwent a reduced-port robotic distal gastrectomy; CLDG, the patients who underwent a conventional laparoscopic distal gastrectomy; C-D grade, grade based on the Clavien–Dindo classification of surgical complications.

## Data Availability

The data presented in this study are available on request from the corresponding author.

## References

[1] Kim H.-I., Han S.-U., Yang H.-K., Kim Y.-W., Lee H.-J., Ryu K.W., Park J.-M., An J.Y., Kim M.-C., Park S. (2016). Multicenter Prospective Comparative Study of Robotic Versus Laparoscopic Gastrectomy for Gastric Adenocarcinoma. Ann. Surg..

[2] Suda K., Man I.M., Ishida Y., Kawamura Y., Satoh S., Uyama I. (2015). Potential advantages of robotic radical gastrectomy for gastric adenocarcinoma in comparison with conventional laparoscopic approach: A single institutional retrospective comparative cohort study. Surg. Endosc..

[3] Obama K., Kim Y.M., Kang D.R., Son T., Kim H., Noh S.H., Hyung W.J. (2018). Long-term oncologic outcomes of robotic gastrectomy for gastric cancer compared with laparoscopic gastrectomy. Gastric Cancer Off. J. Int. Gastric Cancer Assoc. Jpn. Gastric Cancer Assoc..

[4] Lee S., Kim J.K., Kim Y.N., Jang D.-S., Kim Y.M., Son T., Hyung W.J., Kim H.-I. (2017). Safety and feasibility of reduced-port robotic distal gastrectomy for gastric cancer: A phase I/II clinical trial. Surg. Endosc..

[5] Seo W.J., Son T., Roh C.K., Cho M., Kim H.I., Hyung W.J. (2018). Reduced-port totally robotic distal subtotal gastrectomy with lymph node dissection for gastric cancer: A modified technique using Single-Site((R)) and two additional ports. Surg. Endosc..

[6] Lee J.H., Son T., Kim J., Seo W.J., Rho C.K., Cho M., Kim H.-I., Hyung W.J. (2018). Intracorporeal delta-shaped gastroduodenostomy in reduced-port robotic distal subtotal gastrectomy: Technical aspects and short-term outcomes. Surg. Endosc..

[7] Kim Y.Y., Lee Y., Lee C.M., Park S. (2020). Lymphadenectomy using two instrument arms during robotic surgery for gastric cancer: A strategy to facilitate reduced-port robotic gastrectomy. Asian J. Surg..

[8] Kim J.S., Batajoo H., Son T., Choi S., Seo W.J., Cho M., Kim Y.M., Lee J.H., Kim H.-I., Hyung W.J. (2020). Delta-shaped gastroduodenostomy using a robotic stapler in reduced-port totally robotic gastrectomy: Its safety and efficiency compared with conventional anastomosis techniques. Sci. Rep..

[9] Seo W.J., Son T., Shin H., Choi S., Roh C.K., Cho M., Kim H.-I., Hyung W.J. (2020). Reduced-port totally robotic distal subtotal gastrectomy for gastric cancer: 100 consecutive cases in comparison with conventional robotic and laparoscopic distal subtotal gastrectomy. Sci. Rep..

[10] Song J.H., Son T., Lee S., Choi S., Cho M., Kim Y.M., Kim H.-I., Hyung W.J. (2020). D2 Lymph Node Dissections during Reduced-port Robotic Distal Subtotal Gastrectomy and Conventional Laparoscopic Surgery Performed by a Single Surgeon in a High-volume Center: A Propensity Score-matched Analysis. J. Gastric Cancer.

[11] Choi S., Kim N.Y., Kim Y.N., Park S.H., Kim K.-Y., Cho M., Kim Y.M., Hyung W.J., Kim H.-I. (2023). Fluorescence-guided Two-port Robotic Gastrectomy Versus Conventional Laparoscopic Gastrectomy: A Nonrandomized Controlled Trial. Ann. Surg. Open Perspect. Surg. Hist. Educ. Clin. Approaches.

[12] Yang S.C., Ahn J.H., Kim J.H., Yi J.W., Hur M.H., Lee K.Y. (2020). Comparison of the vessel sealer Extend((R)) with harmonic ACE((R)) in robotic bilateral axillary-breast approach thyroid surgery. Gland. Surg..

[13] Lee C.M., Park D.W., Park S., Kim J.H., Park S.H., Kim C.S. (2017). Lymph Node Dissection Using Bipolar Vessel-Sealing Device During Reduced Port Laparoscopic Distal Gastrectomy for Gastric Cancer: Result of a Pilot Study from a Single Institute. J. Laparoendosc. Adv. Surg. Tech. Part A.

[14] In H., Solsky I., Palis B., Langdon-Embry M., Ajani J., Sano T. (2017). Validation of the 8th Edition of the AJCC TNM Staging System for Gastric Cancer using the National Cancer Database. Ann. Surg. Oncol..

[15] Hasson H.M. (1971). A modified instrument and method for laparoscopy. Am. J. Obstet. Gynecol..

[16] Shabbir A., Lee J.H., Lee M.S., Park D.J., Kim H.H. (2010). Combined suture retraction of the falciform ligament and the left lobe of the liver during laparoscopic total gastrectomy. Surg. Endosc..

[17] Japanese Gastric Cancer Association (2017). Japanese gastric cancer treatment guidelines 2014 (ver. 4). Gastric Cancer Off. J. Int. Gastric Cancer Assoc. Jpn. Gastric Cancer Assoc..

[18] Clavien P.A., Barkun J., de Oliveira M.L., Vauthey J.N., Dindo D., Schulick R.D., de Santibañes E., Pekolj J., Slankamenac K., Bassi C. (2009). The Clavien-Dindo classification of surgical complications: Five-year experience. Ann. Surg..

[19] Lee C.M., Park D.W., Jung D.H., Jang Y.J., Kim J.-H., Park S., Park S.-H. (2016). Single-Port Laparoscopic Proximal Gastrectomy with Double Tract Reconstruction for Early Gastric Cancer: Report of a Case. J. Gastric Cancer.

[20] Kim A., Lee C.M., Park S. (2021). Is it Beneficial to Utilize an Articulating Instrument in Single-Port Laparoscopic Gastrectomy?. J. Gastric Cancer.

[21] Lee I.Y., Lee D., Lee C.M. (2023). Case Report: Single-port laparoscopic total gastrectomy for gastric cancer in patient with situs inversus totalis. Front. Oncol..

